# Metastatic progression and treatment challenges in EBV-associated pulmonary lymphoepithelioma-like carcinoma: a case report

**DOI:** 10.3389/fonc.2026.1796167

**Published:** 2026-06-23

**Authors:** Min Wei, Sumin Wu, Yanbo Wang, Mengyuan Zhu, Xiaofen Pan, Chengming Ke

**Affiliations:** 1 Center of Excellence, The Seventh Affiliated Hospital, Sun Yat-sen University, Shenzhen, China; 2 Department of Oncology, The Seventh Affiliated Hospital, Sun Yat-sen University, Shenzhen, China; 3 Neuroscience Medical Center, The Seventh Affiliated Hospital, Sun Yat-sen University, Shenzhen, China

**Keywords:** Epstein-Barr virus, immunotherapy, plasma EBV-DNA, pulmonary lymphoepithelioma-like carcinoma, tumor-related fever

## Abstract

**Objective:**

To describe the clinical characteristics, treatment course, and management challenges of advanced pulmonary lymphoepithelioma-like carcinoma (LELC), with a focus on metastatic disease and treatment-interfering complications.

**Methods:**

We report the clinical course of a 42-year-old woman with EBV-associated pulmonary LELC. The patient was initially diagnosed with stage IIIB disease and underwent radical surgery followed by adjuvant chemoradiotherapy. No definite local recurrence was observed postoperatively; however, cervical lymph node metastasis developed shortly thereafter, followed by rapid progression to stage IV disease characterized predominantly by liver metastases. First-line treatment with pembrolizumab combined with chemotherapy achieved an initial partial response. During subsequent disease progression, the patient developed persistent high-grade fever refractory to antimicrobial therapy, which significantly compromised treatment continuity.

**Results:**

This case demonstrates the potentially aggressive and heterogeneous clinical behavior of pulmonary LELC despite multimodal therapy. Serial plasma EBV-DNA levels closely paralleled disease activity, decreasing during treatment response and increasing during progression. Persistent high-grade fever posed a major diagnostic and therapeutic challenge. Tumor-related fever was considered the most likely etiology, although occult infection could not be definitively excluded. Due to sustained fever, declining performance status, limited clinical experience with immunotherapy at that time, and concerns regarding treatment-related risks, immunotherapy was interrupted during the febrile period.

**Conclusion:**

Advanced pulmonary LELC may exhibit aggressive clinical behavior with predominant metastatic burden. Immunochemotherapy may provide meaningful but often transient benefit in patients with high PD-L1 expression, while dynamic plasma EBV-DNA monitoring may serve as a useful indicator of disease activity. In addition, atypical tumor-related fever may substantially interfere with treatment continuity and clinical decision-making. Early recognition and proactive management of such complications may help optimize outcomes.

## Introduction

Pulmonary lymphoepithelioma-like carcinoma (LELC) is a rare subtype of non-small cell lung cancer (NSCLC) that is closely associated with Epstein–Barr virus (EBV) infection and differs substantially from conventional lung adenocarcinoma and squamous cell carcinoma in terms of epidemiology, pathology, and molecular characteristics (1, 2). Its incidence demonstrates marked geographic variation, being considerably more common in East and Southeast Asian populations than in Western countries (3). Histologically, pulmonary LELC is characterized by undifferentiated carcinoma cells accompanied by dense lymphocytic infiltration, closely resembling nasopharyngeal carcinoma (NPC). This similarity reflects a shared viral etiology and overlapping immune-related molecular features, including activation of inflammatory pathways and frequent programmed death-ligand 1 (PD-L1) expression (4).

Clinically, pulmonary LELC often occurs in younger, non-smoking patients and may initially present with nonspecific symptoms (2, 3). Although early-stage disease is generally associated with relatively favorable outcomes, increasing evidence suggests that locally advanced or metastatic pulmonary LELC may exhibit highly heterogeneous clinical behavior, with some patients experiencing rapid recurrence and aggressive distant metastasis (2, 5, 6). At the molecular level, pulmonary LELC rarely harbors classic actionable driver mutations such as EGFR, ALK, or ROS1 alterations, limiting the applicability of targeted therapies (7). In contrast, high PD-L1 expression and an immune-rich tumor microenvironment are frequently observed, providing a strong biological rationale for the use of immune checkpoint inhibitors (4, 8).

However, because pulmonary LELC is extremely rare, prospective randomized clinical trials are lacking, and current treatment strategies are largely extrapolated from standard NSCLC protocols, therapeutic experience in NPC, and small retrospective studies (2, 9). Recent evidence suggests that PD-1/PD-L1 inhibitor–based immunochemotherapy may provide encouraging clinical benefit in patients with advanced disease, particularly those with high PD-L1 expression (7, 8, 10). In addition, plasma EBV-DNA has become an established biomarker for disease monitoring and prognostic assessment in NPC, and emerging evidence indicates that dynamic EBV-DNA levels may also correlate with tumor burden and treatment response in pulmonary LELC (11). Nevertheless, the optimal duration and continuity of immunotherapy, as well as the management of treatment-interfering complications such as persistent fever, remain poorly defined in clinical practice.

Here, we report a case of EBV-associated advanced pulmonary LELC characterized by early distant metastasis without definite local recurrence, a transient response to pembrolizumab-based immunochemotherapy, dynamic plasma EBV-DNA changes, and persistent high-grade fever that significantly interfered with treatment continuity. This case highlights the aggressive and heterogeneous nature of metastatic pulmonary LELC and underscores potential importance of treatment continuity.

## Case presentation

A 42-year-old female with no significant past medical history presented for a routine physical examination on July 20, 2018. A chest CT showed a 5.6 cm × 4.2 cm mass in the left lower lobe along with several enlarged lymph nodes in the mediastinal and paratracheal regions.

On August 2, 2018, the patient underwent a left lower lobectomy. Postoperative pathological examination confirmed a pulmonary malignancy. Immunohistochemistry (IHC) results showed diffuse strong positivity for CK, p63, p40, and CK5/6, while CK7, CK10/13, TTF-1, Napsin A, and p53 were negative. *In situ* hybridization (ISH) for EBERs was positive. Based on the histomorphology, immunophenotype, and molecular pathology, the diagnosis was consistent with Pulmonary Lymphoepithelioma-like Carcinoma (LELC). Further testing indicated PD-1 negative, PD-L1 expression >50% (negative control was negative), and equivocal ALK (D5F3) expression (negative control was negative). Metastasis was confirmed in 6 out of 25 dissected lymph nodes, involving the subcarinal, contralateral subcarinal, para-esophageal, and dorsal segment nodes. The postoperative pathological stage was pT3N2M0 (Stage IIIB). Genetic testing revealed no mutations in EGFR (exons 18–21) and negative ALK fusion.

From September to October 2018, the patient received postoperative adjuvant concurrent chemoradiotherapy (intensity-modulated radiation therapy [IMRT] with concurrent etoposide and cisplatin). However, only two months after completing adjuvant therapy (December 2018), re-examination revealed metastasis to the left supraclavicular lymph nodes. A left neck lymph node dissection was performed, and pathology reconfirmed metastatic LELC. Postoperatively, supplementary radiotherapy was administered to the bilateral supraclavicular and cervical lymph node drainage areas.

In late March 2019, the patient was admitted to our hospital; however, as the initial surgery and pathological evaluation of the primary lung lesion were performed at an outside institution, the original imaging data and complete pathological documentation were not fully available. However, the resected specimen was subsequently reviewed at our institution, confirming the diagnosis of pulmonary lymphoepithelioma-like carcinoma. Contrast-enhanced CT of the chest and abdomen demonstrated postoperative changes of lymphoepithelioma-like carcinoma in the left lower lobe (**Figures 1a, b**), along with multiple hepatic metastases (**Figure 2a**). The clinical stage was updated to Stage IV (T3N2M1). Serum tumor marker CA125 rose to 65.13 U/ml, and plasma EBV-DNA load reached 9.40×10³ copies/ml. Starting March 30, 2019, the patient began first-line treatment with pembrolizumab combined with nab-paclitaxel and S-1. Assessments after 2 and 4 cycles showed a partial response (PR) (**Figure 2b**), with CA125 decreasing to 42.17 U/ml and EBV-DNA dropping below 5×10² copies/ml. However, after completing 6 cycles, imaging revealed an increase in the size of liver metastases, indicating progressive disease (PD) (**Figure 2c**).

**Figure 1:**
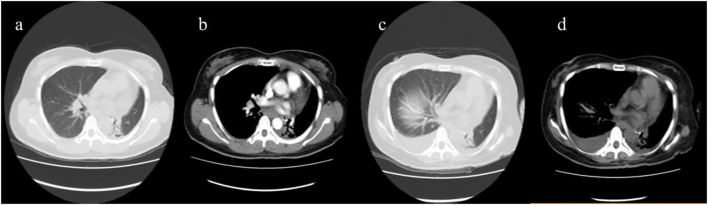
Chest CT images. **(a)** Lung window on March 27, 2019, before treatment following the detection of liver metastasis, showing postoperative changes of lymphoepithelioma-like carcinoma in the left lower lobe. **(b)** Corresponding mediastinal window. **(c)** Lung window on January 3, 2020, during continued disease progression, demonstrating pleural effusion. **(d)** Corresponding mediastinal window.

**Figure 2:**
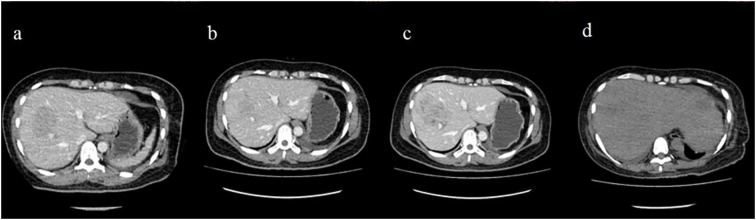
Dynamic radiological changes of liver metastases during the disease course. **(a)** Baseline CT before initiation of pembrolizumab-based immunochemotherapy (March 27, 2019), showing multiple liver metastases. **(b)** Partial response after 4 cycles of immunochemotherapy (June 20, 2019). **(c)** Disease progression after 6 cycles, with enlargement of liver metastases (August 12, 2019). **(d)** Further rapid progression of liver metastases before death (January 3, 2020).

On August 13, 2019, an ultrasound-guided biopsy of the liver lesion was performed. Pathology showed tumor cells expressing CK, p63, p40, CK5/6, and CK19, with weak positivity for Hepatocyte and negative results for CK7, TTF-1, Napsin A, and Arginase-1. EBERs ISH was positive. Consistent EBV positivity was observed in both the reviewed primary lung specimen and the liver metastasis biopsy. Combined with the clinical history, this confirmed LELC liver metastasis. PD-L1 (SP142) expression was observed in approximately 70% of tumor cells (**Figure 3**).

**Figure 3:**
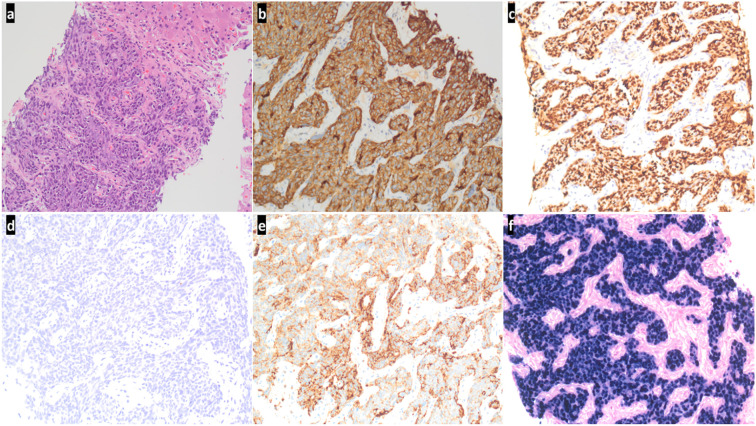
Pathological slides from the primary lung tumor were not available, as the surgery was performed at an outside institution; however, the diagnosis was confirmed through pathological consultation. Representative histopathological and immunohistochemical findings from the liver metastasis are therefore presented. **(a)** Hematoxylin and eosin (H&E) staining shows poorly differentiated carcinoma with prominent lymphoid stroma. **(b)** Tumor cells are diffusely positive for cytokeratin (CK). **(c)** Strong nuclear positivity for p63 is observed in tumor cells. **(d)** Tumor cells are negative for thyroid transcription factor-1 (TTF-1). **(e)** Programmed death-ligand 1 (PD-L1, SP142) shows positive staining in approximately 70% of tumor cells. **(f)** Epstein–Barr virus-encoded RNA (EBER) *in situ* hybridization (ISH) demonstrates positive signals in tumor cells.

Beginning August 23, 2019, the patient developed recurrent high fever without obvious triggers, peaking at 41 °C. Laboratory tests showed a normal white blood cell count and slightly elevated procalcitonin (PCT, 0.16 ng/ml). Multiple blood, urine, stool, and sputum cultures were negative, and imaging revealed no definitive infectious focus. Metagenomic next-generation sequencing (mNGS) of peripheral blood detected sequences of Pantoea, EBV, and Cytomegalovirus (CMV). Despite empiric treatment with broad-spectrum antibiotics (covering bacteria and fungi), corticosteroids (methylprednisolone), and immune support (thymalfasin, intravenous immunoglobulin), the fever persisted. Anti-tumor therapy was interrupted during this period due to the fever and infection workup.

On September 6, 2019, a whole-body PET/CT demonstrated mildly increased metabolic activity in the bronchial stump, likely reflecting postoperative inflammatory changes, along with metabolically active multiple liver metastases. The PET/CT examination was performed at an outside institution, and the original imaging data were not available for inclusion in this report. Imaging assessment confirmed disease progression. On September 9, 2019, a regimen of cetuximab combined with docetaxel and cisplatin was attempted, but only one cycle was completed due to Grade IV myelosuppression and Grade III diarrhea. Subsequent attempts with “gemcitabine combined with oxaliplatin” and “bevacizumab combined with fluorouracil and anlotinib” were poorly tolerated. The patient gradually developed right upper quadrant pain, abdominal distension, and new ascites. A CT scan in November 2019 showed continuous, rapid progression of liver metastases with significant hepatomegaly. Plasma EBV-DNA load rebounded to 7.21×10³ copies/ml.

In early January 2020, the patient’s condition deteriorated sharply with signs of liver failure. Imaging showed hepatomegaly, progression of liver metastases in both size and number (**Figure 2d**), and a small right-sided pleural effusion (**Figures 1c, d**); cytology was negative for malignant cells. On January 10, 2020, as a palliative attempt undertaken solely at the persistent request of the patient’s family, a single dose of nivolumab was administered. However, the patient’s general status was extremely poor; the treatment failed to reverse the clinical course, and she passed away on January 15, 2020, due to multiple organ failure.

## Discussion

Pulmonary lymphoepithelioma-like carcinoma (LELC) is a rare subtype of non-small cell lung cancer (NSCLC) closely associated with Epstein–Barr virus (EBV) infection and differs substantially from conventional lung adenocarcinoma and squamous cell carcinoma in terms of epidemiology, pathology, and clinical behavior (1, 2, 5). Although pulmonary LELC is often considered to have a relatively favorable prognosis (12), this case highlights a distinct and highly aggressive clinical phenotype characterized by early distant metastasis without definite local recurrence and rapid systemic progression.

The aggressive behavior of pulmonary LELC may be partly attributed to its EBV-driven tumor microenvironment. EBV latent proteins, including LMP1 and LMP2A, can activate oncogenic signaling pathways such as NF-κB and JAK/STAT, thereby promoting tumor proliferation, immune evasion, and chronic inflammatory responses (13, 14). Meanwhile, pulmonary LELC frequently demonstrates abundant lymphocytic infiltration and high PD-L1 expression, providing a biological rationale for immune checkpoint inhibitor therapy. Due to the lack of prospective randomized trials, treatment strategies for pulmonary LELC are largely extrapolated from NSCLC and nasopharyngeal carcinoma treatment paradigms (2, 9). Previous retrospective studies and case series have suggested promising activity of PD-1/PD-L1 inhibitor–based immunochemotherapy in patients with high PD-L1 expression (8, 15). In the present case, pembrolizumab combined with chemotherapy achieved an initial partial response, indicating that immunochemotherapy may provide meaningful clinical benefit in selected patients with advanced pulmonary LELC.

Another notable finding in this case was the dynamic change in plasma EBV-DNA levels throughout the disease course. Serial monitoring demonstrated that EBV-DNA levels closely paralleled disease activity, decreasing during treatment response and increasing again during disease progression. Similar to its established role in nasopharyngeal carcinoma (16), these findings suggest that plasma EBV-DNA may serve as a useful noninvasive biomarker for monitoring tumor burden and treatment response in pulmonary LELC.

The management of persistent fever represented the most significant clinical challenge in this case. The patient developed persistent high-grade pyrexia reaching 41 °C, which differed from the typical low-to-moderate fever pattern usually associated with tumor-related fever. The Pantoea species, EBV, and CMV sequences detected via mNGS require cautious interpretation. Pantoea is a common environmental commensal bacterium; given the negative blood culture results, its detection was more likely attributable to contamination or colonization rather than true infection. The detection of EBV was consistent with the virological background of pulmonary LELC and likely reflected tumor-associated viral load. The detection of CMV suggested possible latent viral reactivation; however, the patient showed no clinical or radiological evidence of CMV-related organ disease. Overall, mNGS failed to identify a definitive pathogen that could explain the persistent high-grade fever. Nevertheless, these findings highlight the importance of remaining vigilant for opportunistic infections or viral reactivation following chemo-immunotherapy.

Considering the ineffectiveness of antimicrobial therapy, the temporal correlation between fever and tumor progression, and the lack of definitive microbiological evidence, tumor-related fever was considered the most likely explanation. Tumor-related fever is generally thought to result from pyrogenic cytokines released by tumor cells or the tumor-associated inflammatory microenvironment (17). However, at the time of treatment (2019), clinical experience with immune checkpoint inhibitors was limited, and diagnostic uncertainty remained. In addition, the severity of fever, progressive deterioration in performance status, and concerns regarding treatment-related risks ultimately contributed to the decision to discontinue immunotherapy during the febrile period. Although a direct causal relationship between pembrolizumab interruption and subsequent disease progression cannot be established, this case suggests that treatment continuity may be an important factor in disease control in selected patients.

This study has several limitations. First, as a single-case report, the findings cannot be generalized. Second, the initial imaging studies and complete pathological documentation of the primary lung lesion were not fully accessible, as the surgery was performed at an outside institution. Although the diagnosis was supported by pathological consultation of the resected specimen and confirmed by liver metastasis biopsy, the lack of complete baseline data may limit the interpretation of disease origin and EBV status. Third, PET/CT imaging during disease progression was performed externally, and the original images could not be retrieved. To partially address this limitation, we supplemented chest CT images obtained at our institution at key time points. Finally, treatment decisions were influenced by real-world factors, including limited clinical experience with immunotherapy at that time, persistent fever of uncertain etiology, and patient-related considerations. The administration of a single dose of nivolumab represented a compassionate palliative attempt rather than a protocol-driven therapeutic strategy, and its lack of efficacy should be interpreted in the context of end-of-life care.

In conclusion, pulmonary LELC is a rare EBV-associated malignancy with heterogeneous and potentially aggressive clinical behavior. This case highlights the potential benefit of immunochemotherapy, the clinical value of dynamic plasma EBV-DNA monitoring, and the substantial impact of atypical tumor-related fever on treatment continuity and clinical decision-making. Careful evaluation of persistent fever and proactive management of treatment-interfering conditions may help optimize outcomes in patients with advanced pulmonary LELC.

## Data Availability

The original contributions presented in the study are included in the article/supplementary material. Further inquiries can be directed to the corresponding authors.
